# Characterization of 260 Isolates of *Aspergillus* Section Flavi Obtained from Sesame Seeds in Punjab, Pakistan

**DOI:** 10.3390/toxins14020117

**Published:** 2022-02-04

**Authors:** Maryam Ajmal, Ahmad F. Alshannaq, Heungyun Moon, Dasol Choi, Abida Akram, Brian Gagosh Nayyar, John G. Gibbons, Jae-Hyuk Yu

**Affiliations:** 1 Department of Botany, Faculty of Sciences, Pir Mehr Ali Shah Arid Agriculture University, Rawalpindi 46300, Pakistan; maryamajmal92@yahoo.com (M.A.); abidaakram@uaar.edu.pk (A.A.); 2 Department of Bacteriology, Food Research Institute, University of Wisconsin-Madison, 1550 Linden Drive, Madison, WI 53706, USA; alshannaq@wisc.edu (A.F.A.); harrison.moon@wisc.edu (H.M.); dasol.choi@wisc.edu (D.C.); 3 Department of Botany, Faculty of Sciences, University of Sialkot, Sialkot 51310, Pakistan; brian_gagosh@hotmail.com; 4 Department of Food Science, College of Natural Sciences, University of Massachusetts, Amherst, MA 01003, USA; jggibbons@umass.edu; 5 Department of Systems Biotechnology, Konkuk Institute of Science and Technology, Konkuk University, Seoul 05029, Korea

**Keywords:** sesame, *Aspergillus flavus*, aflatoxins, Pakistan, HPLC, genome analyses

## Abstract

Sesame *Sesamum indicum* L. is a major oil-based seed crop that has been widely cultivated and consumed in Pakistan. Unfortunately, sesame is highly prone to *Aspergillus* fungal growth in the field, and under inappropriate storage conditions can become contaminated with aflatoxins, the most potent carcinogen found in nature. Here, we have isolated a high number of *Aspergillus* isolates from sesame seeds in fresh and stored conditions obtained from rainfed and irrigated zones of Punjab, Pakistan, and characterized them for aflatoxigenic potentials. Using morphological identification techniques, 260 isolates were grouped as potential *Aspergillus* section Flavi, with 126 and 134 originating from the rainfed and irrigated zones, respectively. Out of 260 in total, 188 isolates were confirmed to produce aflatoxins. There were no significant differences in potential aflatoxigenic isolates with respect to the rainfed and irrigated zones. However, the number of potential aflatoxigenic isolates was significantly higher (*p* < 0.05) in stored samples than that of those from fresh sesame seeds in the rainfed and irrigated zone. Whole genome sequencing and comparative analyses of 12 select isolates have revealed that one of the *A. flavus* isolates, which produced very low aflatoxins (AFP10), has an elevated missense variant rate, numerous high impact mutations, and a 600 base pair deletion in the *norB* gene. In summary, our study provides insights into aflatoxigenic potential and the associated genetic diversity of indigenous *Aspergillus* section Flavi isolates and potential management strategies for reducing aflatoxin contamination levels in a major crop consumed in Punjab, Pakistan.

## 1. Introduction

Aflatoxins are a group of naturally occurring carcinogens and toxic secondary metabolites of the fungal genus *Aspergillus* [1]. Numerous *Aspergillus* spp. produce aflatoxins but *Aspergillus flavus* is the most predominant one responsible for producing aflatoxins in fields and storage conditions [2]. The four major aflatoxins commonly isolated from foods and feeds are aflatoxin B_1_ (AFB_1_), aflatoxin B_2_ (AFB_2_), aflatoxin G_1_ (AFG_1_), and aflatoxin G_2_ (AFG_2_), which are categorized as group 1 human carcinogens by the International Agency for Research on Cancer (IARC) [3]. AFB_1_ is the most toxic, common, and widespread food and feed contaminant and is responsible for 75% of all aflatoxin contamination [4]. Over half of the global population (some four billion people), mostly in developing countries, are at threat of chronic exposure to unknown levels of aflatoxins, which can be associated with decreased growth rate and feeding efficiency, reduced liver and kidney function, and immune system suppression [5,6]. The most important illness related to aflatoxin consumption is hepatocellular carcinoma, which is the third leading cause of cancer-related mortality worldwide [7]. In the developing countries, 40% of human productivity is lost as a result of disorders associated with aflatoxin contamination [8]. 

Sesame, *Sesamum indicum* L., is a major oilseed crop that is cultivated in about 9,398,770 hectares (ha) of tropical, sub-tropical, and temperate regions around the world. Pakistan ranks 14th among the major sesame-producing countries in the world with an annual production of about 35,699 tons, grown over 83,372 ha [9]. Sesame is grown in both rainfed and irrigated zones of the Punjab [10]. Sesame represents itself as a rich food source due to its extraordinary oil content ranging from 34% to 59% and its nutritious, medicinal, and cosmetics qualities [11]. Due to the presence of various bioactive compounds, sesame seeds have been shown to exhibit anti-inflammatory, antioxidant, antihypertensive, wound healing, anticancer, and neuroprotective activities [12,13,14,15,16,17]. In Pakistan, sesame seed is a regular part of the local cuisine and is commonly used in the food industry and in Unani herbal medicines. Sesame seeds and sesame oil are used for cooking, salad dressing, garnish, snacks, flavoring agents, as well as for use in the manufacturing of margarine and as a raw ingredient for paints, varnishes, soaps, perfumes and insecticides [18]. Sesame consumption is steadily increasing in Pakistan as well as globally mainly due to changing consumer consumption patterns and increasing health awareness [19]. Sesame is the only oilseed crop exported by Pakistan. During 2019–2020, 32,838 tons of sesame seeds were exported from Pakistan with huge foreign revenue [20]. Sesame is important in Pakistan because of its multidimensional uses. However, contamination from fungal agricultural pests is a major concern [21]. *A. flavus* is the most prevalent aflatoxin producer [22]. The warm and humid conditions of Pakistan, especially in the province of the Punjab, facilitate the thriving of *A. flavus*, and consequently, aflatoxin contamination poses serious threats to the health of the Pakistani people [23,24]. Additionally, many Pakistani farms do not utilize proper handling practices or have proper food storage facilities. As a consequence, economic losses due to *A. flavus* contamination can reach up to 100% when the presence of aflatoxins is beyond acceptable levels [25,26,27].

Aflatoxin production in *A. flavus* is polymorphic, and the collective species are relatively genetically diverse [28,29,30,31,32]. The ability of *A. flavus* to produce aflatoxins is strain-specific and it is associated with the intactness of the aflatoxin biosynthesis gene cluster, which consists of 26 genes responsible for the biosynthesis and transport of aflatoxins [33,34]. Currently, there is increasing awareness of aflatoxin contamination in foods and feeds, and previous studies have reported the presence of *A. flavus* in sesame from Pakistan. However, no systemic studies have been carried out for the aflatoxigenic potential and genetic intactness of the aflatoxin gene cluster in the indigenous *A. flavus* population isolated from sesame seeds in Pakistan. Here, we assessed the contamination rates of *A. flavus* in sesame seeds, determined the aflatoxigenic and non-aflatoxigenic potential of 260 isolates, and carried out comparative genomic analyses of 12 select indigenous *A. flavus* isolates from sesame seeds produced in two Agro-ecological zones of the Punjab, Pakistan.

## 2. Results 

### 2.1. Sesame Seed Samples and Isolation of Fungi Belonging to Aspergillus Section Flavi

**Figure 1:**
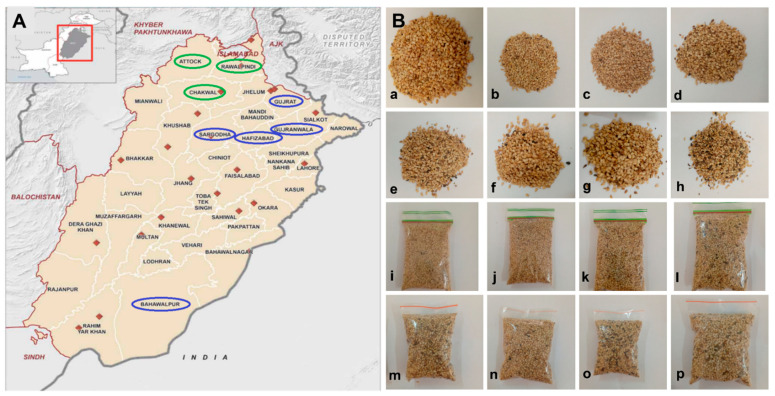
Sample collection regions in Punjab and sesame seed samples. (**A**) A map of Punjab, Pakistan, showing the regions for collection of the sesame seed samples. Green circles indicate the districts of the rainfed, and blue circles indicates the districts of the irrigated zone. (**B**) Fresh sesame seeds collected from (**a**) Rawalpindi, (**b**) Attock, (**c**) Chakwal, (**d**) Hafizabad, (**e**) Gujranwala, (**f**) Gujrat, (**g**) Sargodha, (**h**) Bahawalpur, and (**i**–**p**) the sesame seeds from (**a**) Rawalpindi ~ (**h**) Bahawalpur being stored in polythene bags at room temperature.

One hundred sesame seeds samples were collected directly from the fields in two Agro-ecological zones of Punjab, Pakistan i.e., the rainfed zone (Rawalpindi, Attock and, Chakwal) and the irrigated zone (Hafizabad, Gujranwala, Gujrat, Sargodha, and Bahawalpur: Figure 1A). Representative fresh and stored sesame seed samples used for *Aspergillus* isolation are shown in Figure 1B. 

**Figure 2:**
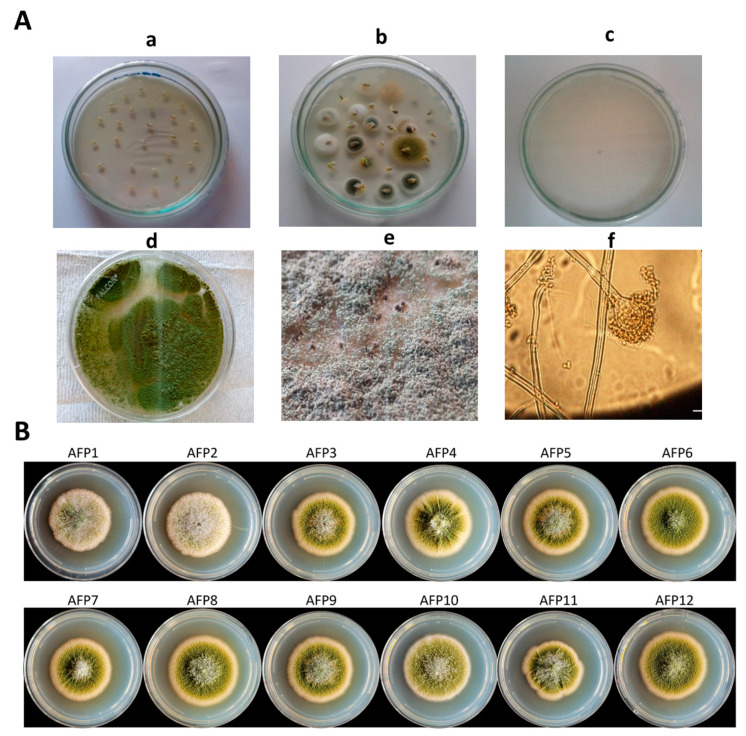
Isolation and identification of fungal isolates belonging to *Aspergillus* section Flavi. (**A**) Procedure for isolation of *Aspergillus* section Flavi from sesame seeds (**a**) sesame seeds were placed on PDA, (**b**) fungal colonies emerging from the seeds after 3–5 days at 28 ± 2 °C under alternative cycle of darkness and light in a versatile environmental test chamber, (**c**) isolation of potential *Aspergillus* section Flavi on the basis of colony characteristics, (**d**) Growth of *Aspergillus* section Flavi on PDA, (**e**) Morphological appearance of *Aspergillus* section Flavi and brown exudates, and (**f**) A microscopic image of conidiophore of *Aspergillus* section Flavi. (**B**) Colonies of the 12 isolates of *Aspergillus* section Flavi selected for whole genome sequencing. Each isolate was point inoculated on the center of PDA and the colony photographs were taken at four days post incubation at 30 °C. Initially, the isolates exhibited the white color of mycelia followed by production of yellow green to olive-colored conidia within three days.

Isolates of *Aspergillus* section Flavi show rapid growth on Potato Dextrose Agar (PDA). Initially, the isolates exhibited the white color of mycelia followed by the production of yellow green to olive-colored conidia within three days of incubation. The reverse side of the colonies was slightly pale. Microscopic examinations showed that the conidiophores were thick walled, non-septate, uncolored and unevenly pitted or roughened vesicles. The length of the conidiophore was about 600–800 µm and the diameter was 15–20 µm. The vesicles were sub-globose in a few isolates and globose in others. Phialides were either uniseriate, biseriate, or both. The conidia were globose with thin, slightly roughened walls. The length of the conidial head was about 20–45 µm yellow/greyish green and the diameter ranged from 2–6 µm (Figure 2A,B).

Out of all the molds in fresh and stored sesame seeds, 260 isolates were identified as potential *Aspergillus* section Flavi based on macro-morphological and micro-morphological characteristics. The present study tested the hypothesis that differences exist between fresh and stored sesame seeds derived from rainfed and irrigated zones in terms of contamination with *Aspergillus* section Flavi. Isolation frequency (%) and relative density (%) of *Aspergillus* section Flavi were calculated and are presented in Table 1.

In the rainfed zone, 126 isolates were identified as potential *Aspergillus* section Flavi including 34 isolates from fresh and 92 isolates from stored sesame seeds. It appears that stored samples were more contaminated with *Aspergillus* than fresh samples with a percentage incidence of about 100% and 80%, respectively. Stored samples from all the collection sites showed 100% occurrence of *Aspergillus* section Flavi while in fresh sesame seeds, and the highest incidence was reported from Rawalpindi (100%) followed by Attock (80%), and Chakwal (60%) (Table 1).

In the irrigated zone, 134 isolates were identified as potential *Aspergillus* section Flavi, which include 56 from fresh and 78 from stored sesame seeds. Stored samples were more contaminated than fresh samples, with the percentage incidence of about 93.32% and 71.84%, respectively. Stored samples from all the collection sites showed 100% occurrence except for Bahawalpur with 66.6% incidence. In the case of fresh samples, the highest incidence was reported from Sargodha and Gujranwala with 100% occurrence and the least was reported from Gujrat with 40% incidence (Table 1).

### 2.2. Examination of the Aflatoxigenic Potential of 260 Aspergillus Section Flavi Isolates

**Figure 3:**
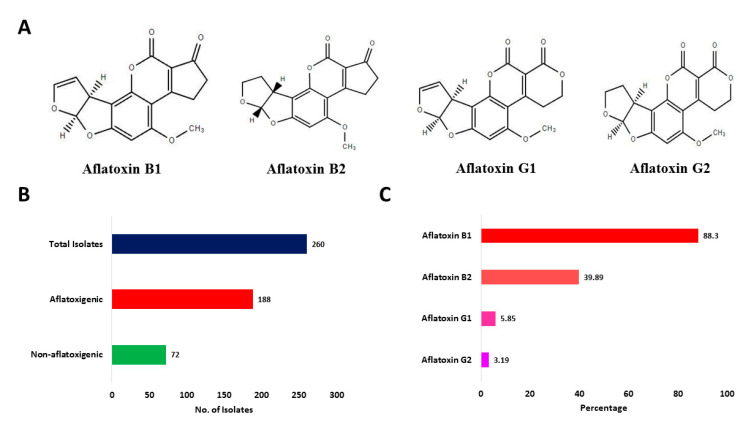
Aflatoxin production potential of *Aspergillus* section Flavi isolates. (**A**) Chemical structures of AFB_1_, AFB_2_, AFG_1_, and AFG_2._ (**B**) Total number of aflatoxigenic and non-aflatoxigenic isolates of *Aspergillus* section Flavi isolated from fresh and stored sesame seeds. (**C**) Percentage of aflatoxigenic isolates producing AFB_1_, AFB_2_, AFG_1_, and AFG_2_.

We then tested all 260 potential Aspergillus section Flavi isolates for their ability to produce aflatoxins in vitro employing HPLC. As shown in Table 2, 188 (72.31%) isolates produced aflatoxins whereas no aflatoxins were detectable from cultures of 72 (27.69%) isolates (Figure 3A,B). From aflatoxigenic isolates, 88.3% isolates were reported to produce AFB_1_, 39.89% isolates produced AFB_2_, 5.85% isolates produced AFG_1,_ and 3.19% isolates were reported to produce AFG_2_ (Figure 3C). The average and range of AFB_1_, AFB_2_, AFG_1_, and AFG_2_ are presented in Table 3. HPLC chromatograms of select *A. flavus* isolates are shown in Supplementary Figure S1. 

In the rainfed zone, 92 (73.02%) isolates were found to be aflatoxigenic (20 isolates from fresh and 72 isolates from stored sesame seeds). However, no aflatoxins were detected in the cultures of 34 (26.98%: 14 isolates from fresh and 20 isolates from stored sesame seeds). The chances of getting aflatoxigenic isolates of *Aspergillus* section Flavi were significantly (*p* < 0.05) higher in stored samples as compared to fresh samples with frequencies of 78.26% and 58.82%, respectively. Moreover, the occurrence of aflatoxigenic *Aspergillus* section Flavi isolates was significantly (*p* < 0.05) higher in stored samples compared to fresh samples in all collection sites including Rawalpindi, Attock, and Chakwal (Table 2).

In the irrigated zone, 96 (71.64%) isolates were found to be aflatoxigenic (32 isolates from fresh and 64 isolates from stored sesame seeds) and 38 (28.36%) isolates were reported as non-aflatoxigenic (24 isolates from fresh and 14 isolates from stored sesame seeds). The occurrence of aflatoxigenic *Aspergillus* section Flavi isolates was significantly (*p* < 0.05) higher in stored samples as compared to fresh samples with the frequency of 82.05% and 57.14%, respectively. Moreover, the chances of getting aflatoxigenic *Aspergillus* section Flavi isolates was significantly (*p* < 0.05) higher in stored samples compared to fresh samples in Gujranwala, Gujrat, Sargodha, and Bahawalpur, except for Hafizabad (*p* ˃ 0.05) (Table 2).

### 2.3. Whole Genome Sequencing and Comparative Analyses of 12 Pakistani Isolates 

To understand the genetic and genomic bases of the differences in their aflatoxigenic potential, 12 isolates (see Figure 2B) of *Aspergillus* section Flavi were chosen for the whole-genome sequencing analyses. As shown in Table 4, we selected six high producers of aflatoxins (AFP1~AFP6), and three were medium aflatoxin producers (AFP7~AFP9) and three were very low aflatoxin producers (AFP10~AFP12). Whole-genome sequencing data were deposited on the NCBI SRA under “*Aspergillus flavus*, Pakistani isolates Genome sequencing” (Accession No. PRJNA682409). Each genome was sequenced to a coverage of 25x~37x. The genome sequences of all 12 isolates highly matched to that of the reference *A. flavus* NRRL3357, confirming that all these isolates belong to *A. flavus* species (Supplementary Table S1). As shown in Figure 2, the 12 isolates have different developmental phenotypes. 

#### 2.3.1. Phylogenetic Analysis

**Figure 4:**
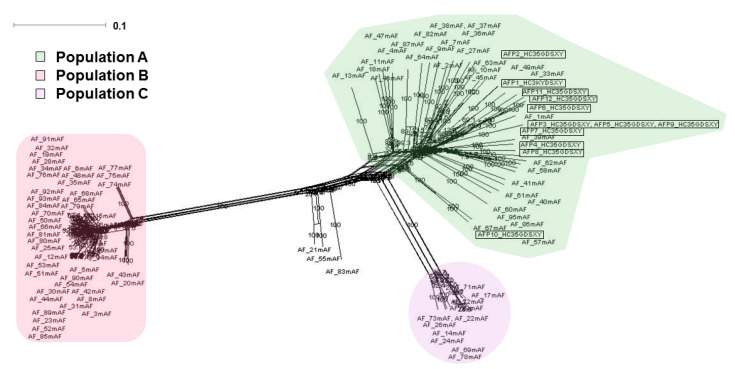
Phylogenetic network analysis of the 12 Pakistani *A. flavus* isolates with 1000 bootstraps along with 94 isolates of *A. flavus*. Neighbor-net phylogenetic network analysis of 3581 SNPs spaced by a minimum of 10 kb revealed three major populations. All Pakistani isolates (marked by box) are nested within population A. Branch lengths suggest that population A has higher levels of genetic diversity.

We inferred the evolutionary history of the 12 Pakistani *A. flavus* isolates along with 94 isolates of *A. flavus* strains from the United States that had been previously sequenced by Drott et al. [31]. Neighbor-net phylogenetic network analysis of 3581 SNPs spaced by a minimum of 10 kb revealed three major populations, corroborating the findings of Drott et al. [31]. All 12 Pakistani isolates are nested within population A (Figure 4). Branch lengths leading to terminal taxa are longer in population A compared with populations B and C, suggesting that population A has higher levels of genetic diversity (Figure 4).

#### 2.3.2. Nucleotide Variation Analysis

**Figure 5:**
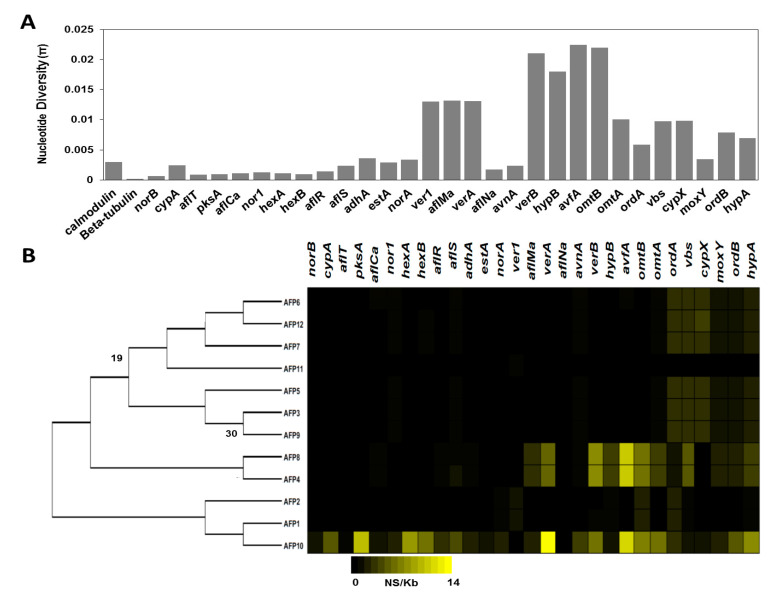
Nucleotide diversity and missense variant rate of 12 isolates. (**A**) Nucleotide diversity (π) in the 12 Pakistani *A. flavus* isolates across the aflatoxin cluster genes and the housekeeping genes calmodulin and beta-tubulin. (**B**) Missense variant rate across genes in the aflatoxin gene cluster. Black represents a missense variant rate of 0, while bright yellow represents a missense variant rate of 14. *A. flavus* isolates are depicted based on their phylogenetic relationship from a maximum likelihood tree. The tree is rooted at the midpoint, and bootstrap values are shown when below 80.

To gain insights into associations between nucleotide variation and aflatoxin production, we calculated nucleotide diversity (π) within the Pakistani isolates across each gene in the aflatoxin gene cluster (*norB − hypA*) and two housekeeping genes encoding *beta-tubulin* and *calmodulin*. The highest levels of nucleotide diversity were present in *avfA* (π = 0.022), *omtB* (π = 0.022), and *verB* (π = 0.021). The lowest levels of nucleotide variation were observed in *beta-tubulin* (π = 0.0002), *norB* (π = 0.0007), *aflT* (π = 0.0009), *pksA* (π = 0.0009) and *fas-1* (π = 0.001) (Figure 5A). Additionally, we quantified the missense variant rate for each isolate in each gene in the aflatoxin cluster as well as the housekeeping genes *beta-tubulin* and *calmodulin*. Only the isolates AFP8, AFP4, and AFP10 had missense variant rates greater than 0.6 (AFP8 = 1.98, AFP4 = 2.01, and AFP10 = 4.13). These isolates all possessed missense variant rates greater than 5 in *avfA*, *verB*, *omtB* and *verA*. AFP10 also displayed missense variant rates higher than 5 in *pksA*, *fas2*, *hypA*, *omtA*, and *fas1* (Figure 5B).

#### 2.3.3. Prediction of High Impact Mutations 

Furthermore, we predicted putative high impact mutations as defined by SnpEff [35] in each isolate in all genes in the aflatoxin gene cluster. Interestingly, only AFP10 possessed SNP-based high impact mutations. AFP10 contained a premature stop codon in *hypB* and *cypA*, as well as a splice donor variant in *cypA* (Table 5). Additionally, AFP1, AFP10, AFP11, AFP2, AFP4 and AFP8 contained INDEL-based high impact mutations in aflatoxin genes. AFP10 possessed five high impact INDEL mutations (in *hypA*, *avfA*, *verA*, *aflT*, and *cypA*) (Table 5). 

#### 2.3.4. Copy Number Variation in the Aflatoxin Cluster Locus

**Figure 6:**
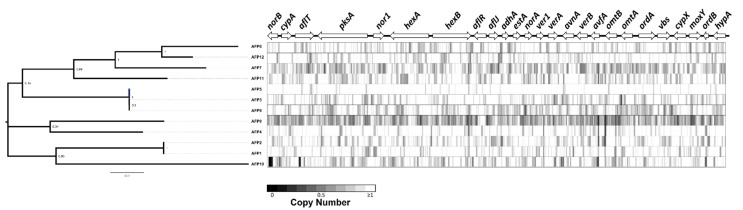
Copy number variation and deletions across the aflatoxin cluster locus of 12 isolates.

Lastly, we predicted copy number variation across non-overlapping 100 bp bins in all isolates across the aflatoxin cluster locus using a read depth approach. This analysis allowed us to identify large-scale deletions. We observed a ~600 bp deletion in *norB* and a smaller deletion in *aflT* in AFP10 (Figure 6). The high missense variant rate, presence of numerous high impact mutations, and presence of large deletions in the aflatoxin gene cluster in AFP10 coincides with very low aflatoxin production.

## 3. Discussions

Microorganisms can grow on different commodities in fields, during harvesting, transportation, and storage. As filamentous fungi (molds) are ubiquitous and their spores can survive for several years in commodities, therefore, some careful measurements should be taken during storage [36]. In the present study, we have demonstrated that the Pakistani sesame seeds derived from Punjab were highly contaminated with the aflatoxigenic isolates of *Aspergillus* section Flavi in fresh and stored conditions. The primary reason for this high incidence of *Aspergillus* section Flavi is that the samples were collected from the areas where average temperature was 23~30 °C, an optimum range for *A. flavus* proliferation [37]. The hot and humid climatic conditions of rainfed and irrigated zones of Punjab, Pakistan, temperature ideal for fungal contamination, coupled with other factors including agronomic practices, post-harvest treatment (processing, drying, storage), and duration of storage may have contributed to the fungal growth, development, and secondary metabolite formation in agricultural products [38,39,40,41,42].

In Pakistan, limited studies have been carried out on the incidence of *A. flavus* in sesame seeds. In agreement with the present study, previous studies reported the aflatoxigenic potential of *A. flavus* and found that *Aspergillus* section Flavi members were common colonizers of all types of sesame seeds during post-harvest storage, [43,44,45,46,47,48,49,50,51,52,53,54,55,56,57,58,59,60,61,62,63,64,65,66,67]. In one study, 17 sesame samples were collected from Plateau State, Nigeria and were investigated for frequency of fungal species, especially for the *Aspergillus* species which revealed the highest frequency of *A. flavus* [68]. Ninety-one isolates of *A. flavus* derived from 63 samples of sesame collected from North Algeria were tested for their ability to produce aflatoxin and 23.52% of strains were aflatoxigenic [69]. *A. flavus* isolated from sesame seeds were aflatoxigenic, as reported by Sabry et al. [70]. In one study, sesame seeds were purchased from local markets of Nigeria and analyzed for fungal and mycotoxin contamination. The molecular analysis reported the presence of *Aspergillus candidus*, *Aspergillus flavus*, *Aspergillus niger*, *Cladosporium* spp., *Fusarium fujikuroi*, *Penicillium* spp., and *Pleosporales/Didymellaceae* spp. in the sesame seeds. The most frequent mycotoxin in the sesame seeds was AFB_1_ with the occurrence of 76% [71]. In the study by Abbas et al. [72], four sesame varieties were planted in the Mississippi Delta at four nitrogen fertilizer application rates from 44.8 to 112 kg N/ha for the evaluation of grain yields and contamination of mycotoxins, which revealed that N fertilizer application rate had no effect on yield or mycotoxin contamination in 2014, but significantly increased yield in 2015. Some studies from Pakistan reported the presence of *A. flavus* in sesame. Sesame seeds were collected from the National Agriculture Research Council and tested for the presence of mycoflora, which revealed that *A. flavus, A. niger*, and *Fusarium oxysporum* were predominant [73]. Sesame seeds from various areas of Sialkot, Pakistan was studied for mycoflora. A total number of 36 species belonging to 10 genera of fungi were isolated. The prevalent genera were *Fusarium, Penicillium, Cercospora, Alternaria, and Cladosporium,* followed by *Aspergillus* [74]. All the above-mentioned studies undoubtedly revealed that sesame is a highly threatened commodity with *A. flavus*. 

In the present study, whole genome analyses have revealed that one isolate (AFP10; a very low aflatoxin producing isolate) has SNP-based high impact mutations, five high impact INDEL mutations (in *hypA*, *avfA*, *verA*, *aflT*, and *cypA*), and a 600 bp deletion in *norB*. Previous studies reported deletions in the *norB*–*cypA* region and several other large deletions in the aflatoxin-biosynthesis gene cluster in *A. flavus* [75,76,77,78,79]. The present study was also supported by previous reports in which 281 isolates of *A. flavus* were tested for aflatoxin production. The population was subdivided into two genetically different populations (A and B) which differ in allelic and genotypic diversity. The less diverse population was more abundant and may represent a clonal lineage derived from more diverse populations [80]. In a study, 94 isolates of *A. flavus* were sampled in the eastern and central latitudinal transects from seven states of the United States. The total population was divided into three genetically distinct populations (A, B, and C), which vary greatly in their ability to generate recombination, diversity, and to produce aflatoxin. Population B is sympatric with population A but produces substantially less aflatoxin and is the only population where multiple gene deletions have clarified the inability of non-aflatoxigenic isolates to produce aflatoxin. Population C is predominantly non-aflatoxigenic [31]. The present study was also confirmed by Adhikari et al. [81] In this study, clusters of aflatoxin genes from 35 genotypes were analyzed, showing a high degree of variations in terms of the amount and size of the gene deletions. Genotypes varied from those with a complete aflatoxin gene cluster to those with no genes at all, with most deletions occurring at the left end of the cluster or towards the telomeric end, depending on the size and type of deletions. In one study, isolates of *A. flavus* were tested for the presence/intactness of the *aflD* and *aflQ* genes. Any mutation or variation in these genes results in low aflatoxin production [82].

## 4. Conclusions

This study provides information on the aflatoxigenic potential and genetic diversity of certain isolates of *Aspergillus* section Flavi obtained from fresh and stored sesame seeds grown in Punjab, Pakistan. Punjab is a major sesame seed producing area of Pakistan and the world. Whole genome analyses of 12 aflatoxigenic isolates have revealed that the highest levels of nucleotide diversity were present in *avfA,* followed by *omtB* and *verb*. AFP10 (very low aflatoxin producing isolate) possessed SNP-based high impact mutations, five high impact INDEL mutations (in *hypA*, *avfA*, *verA*, *aflT*, and *cypA*), and a 600 bp deletion in *norB* and a smaller deletion in *aflT*. The high missense variant rate, the presence of numerous high impact mutations and large deletions in the aflatoxin gene cluster in AFP10 explains very low aflatoxin production. The identification of mutation and deletion in genes significantly affecting the resistance to aflatoxin accumulation would accelerate the development of resistant strains native to local agricultural areas, thus requiring more investigations.

## 5. Materials and Methods

### 5.1. Study Area and Sesame Seeds Samples Collection

The study was carried out in two Agro-ecological zones (rainfed and irrigated) of Punjab, Pakistan. One hundred sesame seeds samples were collected directly from the field in major sesame producing areas of rainfed (Rawalpindi, Attock and, Chakwal) and irrigated (Hafizabad, Gujranwala, Gujrat, Sargodha, and Bahawalpur) zones during the harvest season (Figure 1A). Samples were taken in the Mycology Laboratory, Department of Botany, Pir Mehr Ali Shah Arid Agriculture University Rawalpindi, Pakistan and subjected to the Agar Plate Method for the isolation of Aspergillus section Flavi. After that, sesame seeds samples were stored for 12 months at room temperature (Figure 1B). After 12 months, seeds were again analyzed for Aspergillus section Flavi contamination.

### 5.2. Isolation and Morphological Identification of Aspergillus Section Flavi

Seeds for isolating fungi were surface sterilized with 2% aqueous sodium hypochlorite (NaOCl) for two minutes. Potato Dextrose Agar (PDA, Oxoid, UK) (containing 4 g potato starch, 20 g glucose and 15 g agar) was prepared, 0.1 mg/mL streptomycin was added, and the culture media was poured in to 9 cm diameter petri dishes [83]. About 25 sesame seeds were placed on PDA and incubated for 3–5 days at 28 ± 2 °C under alternative cycles of darkness and light in a versatile environmental test chamber (Sanyo, Japan), with illumination provided by 55 W fluorescent tubes (125–130 μmolm^−2^s^−1^) [84]. The experiment was performed in triplicate. After 3–5 days, seeds were investigated under a light microscope (Eclips 80i, NIKON, Tokyo, Japan), and isolates of *Aspergillus* section Flavi arising from the seeds were counted. Isolates were maintained on fresh PDA and identified based on macro-morphological and micro-morphological characteristics (color and texture of colonies and morphology of conidial head, stipes, hyphal color, conidia size, color, length, shape of vesicle and metula produced on PDA) based on published procedure [46,47,85,86,87]. 

### 5.3. Aflatoxigenic and Non-Aflatoxigenic Potential of Aspergillus Section Flavi

All the isolates of *Aspergillus* section Flavi isolated from fresh and stored sesame seeds were analyzed for their aflatoxigenic and non-aflatoxigenic potential using the methodology described by Alshannaq et al. [88]. Individual aflatoxin standards for AFB_1_, AFB_2_, AFG_1_ and AFG_2_ were purchased from Sigma Chemical Co (St. Louis, MO, USA). As a positive control, the aflatoxigenic strain *A. flavus* NRRL 3357 and as a negative, *A. oryzea* NRRL 2999 was used.

#### 5.3.1. Fungal Culture Preparations and Aflatoxin Extraction 

Isolates of *Aspergillus* section Flavi were cultured into semi-solid (slant), solid and submerged conditions. For the semi-solid (slant) culture, 2 mL of Potato Dextrose Broth (PDB, Difco Lab, Sparks, MD, USA) was added in 25 mL glass test tubes, and fungal isolates (5 × 10^5^ conidia/tube) were inoculated with the help of an inoculating loop. The tubes were put in a rack at a 45°angle and placed in an incubator at 28 ± 2 °C for seven days. For solid culture, fungal isolates (5 × 10^5^ conidia/petri dish) were inoculated in petri dishes containing 25 mL of PDA (Difco Lab, Sparks, MD, USA) and placed in an incubator at 28 ± 2 °C for seven days. For submerged culture, fungal isolates at 5 × 10^5^ conidia/flask were inoculated in 250 mL Erlenmeyer flasks containing 100 mL of PDB. All the flasks were incubated for seven days at 28 ± 2 °C with shaking at 220 rpm. After 7 days of incubation, aflatoxins were extracted from semi-solid, solid, and submerged cultures of fungal isolates as described in Alshannaq et al. [88]. Prior to HPLC analysis, all samples were filtered (0.45 mm with a diameter of 47 mm) (Thermo Fisher Science, Rockwood, TN, USA) into HPLC vials via a PTFE 0.45 μm syringe filter (0.45 mm with a diameter of 17 mm) (Thermo Fisher Science, Rockwood, TN, USA).

#### 5.3.2. HPLC Analysis of Aflatoxins

The samples for AFB_1_, AFB_2_, AFG_1_, and AFG_2_ were analyzed using a Model 1100 HPLC device containing degasser, an autosampler, a quaternary pump fitted with a 1260 Infinity diode array (DAD), and a 1260 Infinity II fluorescence detector (FLD) connected in series (Agilent Technologies, Santa Clara, CA, USA). Samples were monitored for UV detection at a 365 nm wavelength and for FLD detection at 365 nm excitation and 450 nm emission. The samples were eluted with a mobile phase of water (H_2_O)/methanol (CH_3_OH)/acetonitrile (CH_3_CN) (50:40:10) at a flow rate of 0.8 mL/min. Before use, the mobile phase was degassed and purified through a membrane filter (47 mm, 0.45 µm). The injection volume was 100 μL. By using the Chem Station software, peak areas of aflatoxins were obtained and integrated (Agilent Technologies, Santa Clara, CA, USA).

### 5.4. Molecular Characterization

Twelve isolates of *A. flavus* were selected for molecular characterization based on aflatoxin producing ability. Six isolates were high aflatoxin producers, three isolates were medium producers and three were very low aflatoxin producers. The molecular characterization of selected isolates of *A. flavus* was performed by Whole Genome Sequencing (WGS) and their phylogenetic analysis was performed.

#### 5.4.1. DNA Extraction

DNA of *A. flavus* isolates was extracted as described in Lee et al. [89]. DNA was extracted by using a commercially available DNA extraction kit (DNeasy^®^ Plant Mini column kit, Qiagen, UK) by following the manufacturer’s instructions. The presence and quality of pure genomic DNA were confirmed through a 1% (*w*/*v*) agarose gel electrophoresis, and analysis was conducted at 110 V for 35 min. The gel was then viewed under a UV Transilluminator (Biorad Gel DocTM XR + Philadelphia, PA, USA) to confirm the presence of high molecular weight genomic DNA. The eluted DNA was stored at −80 °C until further molecular identification assays.

#### 5.4.2. Genome Sequencing and Quality Control

Illumina libraries were constructed and sequenced by Novogene USA. Illumina sequencing was conducted in a paired-end 150 bp format with a 350 bp insert size. Raw reads were deduplicated using Tally with the “with-quality” and “pair-by-offset” parameters to remove exact duplicates [90]. The number of filtered read pairs and the % of reads that mapped against the *A. flavus* NRRL 3357 reference genome were presented in (supplementary Table S1). Next, deduplicated reads were trimmed with Trim Galore (http://www.bioinformatics.babraham.ac.uk/projects/trim_galore/ accessed on 2 November 2020) using the “stringency 1”, “quality 30” and “length 50” parameters to remove residual adaptor sequences and low-quality positions.

#### 5.4.3. Variant Calling and Annotation

Deduplicated, adapter and quality trimmed paired end reads from the 12 *A. flavus* isolates from Pakistan and the 94 publicly available *A. flavus* genomes previously sequenced by Drott et al. [31] were mapped against the *A. flavus* NRRL 3357 reference genome using BWA-MEM v0.7.15 [91,92]. SNPs and INDELs were called using freebayes v1.3.1 with the default settings with the exception of setting ploidy to haploid (--ploidy = 1) [93]. vcftools v0.1.14 was then used to filter variants with the following parameters: “--remove-filtered-all”, “--minQ 20”, “--recode” and “--recode-INFO-all” [94]. SNPs and INDELS from the filtered VCF file were annotated with SnpEff v4.3t using “*Aspergillus flavus*” as the genome database [35].

#### 5.4.4. Phylogenetic Analysis

We conducted phylogenetic network analysis to infer the evolutionary relationship of the 12 *A. flavus* isolates from Pakistan in relation to the 94 *A. flavus* isolates previously sequenced by Drott et al. [31]. First, we used vcftools to ensure SNP markers were spaced by a minimum of 10 Kb in an effort to limit bias introduced by linkage. After filtering, 3581 SNPs remained, and an alignment of these sites were used to construct the neighbor-net phylogenetic network using Splitstree v4.16.1 [95] with 1000 bootstrap replicates. Additionally, a maximum likelihood phylogenetic tree was constructed in MEGA [96] with the 12 *A. flavus* isolates from Pakistan, using the Tamura-Nei model with 100 bootstrap replicates.

#### 5.4.5. Genomic Analysis of Aflatoxin Loci

First, we used vcftools in haploid mode (“--haploid” option) to calculate nucleotide diversity (π) for each gene in the aflatoxin cluster. Next, using the SnpEff output, we calculated the missense variant rates for each gene in the aflatoxin cluster to identify genes with relatively elevated occurrences of missense variants. The er gene missense variant rate was calculated as:

(1) $$\mathrm{Missense}\;\mathrm{Variant}\;\mathrm{Rate}=\frac{\mathrm{number}\;\mathrm{of}\;\mathrm{missense}\;\mathrm{variants}}{\mathrm{length}\;\mathrm{of}\;\mathrm{all}\;\mathrm{exons}\;\times \;1000}$$

Finally, we used the samtools depth function on sorted bam files to generate coverage values for each site in the aflatoxin locus as described previously by Alshannaq et al. [97]. Average coverage values for each non-overlapping 100 bp portion of the aflatoxin gene cluster were divided by the average coverage across the entire genome to estimate the copy number. Per genome average coverage was estimated by summing the samtools depth output and dividing by the *A. flavus* NRRL 3357 genome size (36,892,344 bp). Bins with coverage values of 0 represent deletions.

### 5.5. Statistical Analysis

Data were summarized and analyzed by using SPSS (version 16.0; SPSS Inc., Chicago, IL, USA). The isolation frequency and relative density of *Aspergillus* section Flavi were calculated by using the following formula.

(2) $$\mathrm{Fr}\;\left(\%\right)=\frac{ns}{N}\times 100$$

(3) $$\mathrm{RD}\left(\%\right)=\frac{ni}{Ni}\times 100$$

where *ns* is the number of samples on which a fungus (*Aspergillus* section Flavi) occurred, *N* is the total number of seed sampled, *ni* is the number of isolates of a fungal genus/species (*Aspergillus* section Flavi), and *Ni* is the total number of fungal isolates obtained. Two-way Analysis of Variance (ANOVA) was performed with significant (*p* < 0.05) to determine the significant difference of aflatoxigenic potential of *Aspergillus* section Flavi isolates among the fresh and stored sesame seed samples obtained from rainfed and irrigated zones of the Punjab, Pakistan.

## Figures and Tables

**Table 1:** Isolation frequency (%) and relative density (%) of *Aspergillus* section Flavi isolates in fresh and stored sesame seeds from two Agro-ecological (rain-fed and irrigated) zones of the Punjab, Pakistan.

Agro-Ecological Zones	Collection Site	Seed Condition	Fr (%)	RD (%)
Rain fed	Rawalpindi	Fresh	100	6.18
		Stored	100	36.36
	Attock	Fresh	80	7.69
		Stored	100	9.82
	Chakwal	Fresh	60	5.10
		Stored	100	20
Irrigated	Hafiz Abad	Fresh	69.2	1.92
		Stored	100	3.87
	Gujranwala	Fresh	100	5.09
		Stored	100	7.85
	Gujrat	Fresh	40	0.89
		Stored	100	14.87
	Sargodha	Fresh	100	10.27
		Stored	100	18.42
	Bahawalpur	Fresh	50	0.99
		Stored	66.6	6.83

Fr = Isolation frequency; RD = Relative Density.

**Table 2:** Aflatoxigenic potential of 260 isolates of *Aspergillus* section Flavi.

Parameters		Seed Conditions	Rainfed				Irrigated					
			Rawalpindi	Attock	Chakwal	Total	Hafizabad	Gujranwala	Gujrat	Sargodha	Bahawalpur	Total
**Aflatoxigenic** **isolates** ***n*** **(%)**		**Fresh**	8 (72.73) **	6 (54.55) *	6 (50.00) *	20(58.82) *	16 (80.00) ***	8 (72.73) **	3 (20) *	3 (50) **	2 (50) **	32 (57.14) **
		**Store**	20 (80.00) ***	22 (73.33) **	30 (81.08) ***	72(78.26) **	20 (80.00) ***	12 (80) ***	16 (80) ***	9 (90) ***	7 (87.5) ***	64 (82.05) ***
**No. of isolates showing AFB1, AFB2, AFG1 and AFG2**	**AFB1**	**Fresh**	8	4	3	15	16	8	3	3	2	32
		**Store**	15	18	27	60	19	11	14	8	7	59
	**AFB2**	**Fresh**	5	4	3	12	3	3	0	2	0	8
		**Store**	8	7	14	29	7	7	7	4	2	27
	**AFG1**	**Fresh**	0	0	0	0	0	0	0	0	0	0
		**Store**	0	2	2	4	2	3	0	2	0	7
	**AFG2**	**Fresh**	0	0	0	0	0	0	0	0	0	0
		**Store**	0	2	2	4	0	0	0	2	0	2
**Non-aflatoxigenic isolates *n* (%)**		**Fresh**	3 (27.27) *	5 (45.45) *	6 (50.00) *	14(41.10) *	4 (20.00) *	3 (27.27) *	12 (80) ***	3 (50) **	2 (50) **	24 (42.85) *
		**Store**	5 (20.00) *	8 (26.67) *	7 (18.92)	20(21.74) *	5 (20.00) *	3 (20.00) *	4 (20) *	1 (10)c	1 (12.5)	14 (17.95)

* (*p* < 0.05), ** (*p* < 0.01), *** (*p* < 0.005).

**Table 3:** Average and range of AFB_1_, AFB_2_, AFG_1_ and AFG_2_ of aflatoxigenic isolates of *Aspergillus* section Flavi in semi-solid (slant) culture condition.

Parameters		Seed Conditions	Rainfed				Irrigated					
			Rawalpindi	Attock	Chakwal	Total	Hafizabad	Gujranwala	Gujrat	Sargodha	Bahawalpur	Total
**Average Aflatoxin (** **µgkg^−1^)**	**AFB1**	**Fresh**	128.31 **	18.25	50.23 *	65.14 *	20.84	55.98 *	23.55	121.13 **	55.29 *	41.67 *
		**Stored**	117.82 *	128.23 **	78.06 *	105.23 **	196.14 **	93.50 *	302.99 ***	20.91	37.64	165.08 **
	**AFB2**	**Fresh**	10.90	8.34	6.98	8.69	5.61	3.55	0.00	5.71	0.00	3.31
		**Stored**	11.42	4.44	12.13	9.43	5.00	13.60	6.65	16.85	8.00	8.90
	**AFG1**	**Fresh**	0.00	0.00	0.00	0.00	0.00	0.00	0.00	0.00	0.00	0.00
		**Stored**	0.00	2.07	1.54	1.29	0.8196	1.41	0.00	4.64	0.00	1.13
	**AFG2**	**Fresh**	0.00	0.00	0.00	0.00	0.00	0.00	0.00	0.00	0.00	0.00
		**Stored**	0.00	1.05	0.99	0.74	0.00	0.00	0.00	3.92	0.00	0.50
**Range of Aflatoxin** **(** **µgkg^−1^)**	**AFB1**	**Fresh**	1.48–361.20	6.20–75.33	8.90–363.26	1.48–363.2	6.50–34.22	5.39–135.01	4.89–180.70	11.33–405.50	20.70–200	4.89–405.57
		**Stored**	2.78–357.60	49.97–350	3.74–171.10	2.78–357.6	21.84–616.40	2.72–367.94	9.43–2503.10	15.34–48.95	8.25–75.5	2.72–2503.1
	**AFB2**	**Fresh**	15.00–33.00	8.91–28.60	2.00–50.78	2.00–50.78	10.27–56.13	3.52–20.27	–	5.25–29.03	–	3.52–56.13
		**Stored**	20.00–50.10	10.0–29.57	5.33–64.40	29.50–64.4	15.16–60.25	15.27–40.60	20.22–40.40	30.32–60.20	5.67–58.34	5.67–60.25
	**AFG1**	**Fresh**	–	–	–	–	–	–	–	–	–	–
		**Stored**	–	5.40–56.58	10.60–46.40	5.40–56.58	2.26–18.23	2.28–10.90	–	8.74–37.60	–	2.2–37.64
	**AFG2**	**Fresh**	–	–	–	–	–	–	–	–	–	–
		**Stored**	–	2.23–29.29	10.23–26.70	2.23–29.29	–	–	–	14.11–25.10	–	14.11–25.10

* (*p* < 0.05), ** (*p* < 0.01), *** (*p* < 0.005).

**Table 4:** Molecular identification and accession numbers of the genomes of the 12 isolates.

Isolates Number	Morphological Identification	Aflatoxin Production	Aflatoxin Level (µgkg^−1^)	Molecular Identification	NCBI Accession Number
AFP1	*Aspergillus flavus*	High aflatoxin production	2503.71	*Aspergillus flavus*	SRX9628150
AFP2	*Aspergillus flavus*	High aflatoxin production	1599.62	*Aspergillus flavus*	SRX9630624
AFP3	*Aspergillus flavus*	High aflatoxin production	616.48	*Aspergillus flavus*	SRX9630884
AFP4	*Aspergillus flavus*	High aflatoxin production	405.57	*Aspergillus flavus*	SRX9631392
AFP5	*Aspergillus flavus*	High aflatoxin production	399.26	*Aspergillus flavus*	SRX9631389
AFP6	*Aspergillus flavus*	High aflatoxin production	367.94	*Aspergillus flavus*	SRX9631420
AFP7	*Aspergillus flavus*	Medium aflatoxin production	110.54	*Aspergillus flavus*	SRX9631445
AFP8	*Aspergillus flavus*	Medium aflatoxin production	119.45	*Aspergillus flavus*	SRX9631446
AFP9	*Aspergillus flavus*	Medium aflatoxin production	139.23	*Aspergillus flavus*	SRX9631391
AFP10	*Aspergillus flavus*	Very low aflatoxin production	4.15	*Aspergillus flavus*	SRX9631390
AFP11	*Aspergillus flavus*	Very low aflatoxin production	16.80	*Aspergillus flavus*	SRX9631387
AFP12	*Aspergillus flavus*	Very low aflatoxin production	26.72	*Aspergillus flavus*	SRX9631388

**Table 5:** High impact mutations in all genes in the aflatoxin gene cluster in 12 isolates.

SNPs							
Isolate	Annotation	Gene_id	CHR	SITE	REF	ALT	Type
AFP10	aflLa/hypB	AFLA_139240	EQ963478	2205372	C	T	stop gained
AFP10	aflU/cypA/P450 monooxygenase	AFLA_139430	EQ963478	2252122	A	G	splice donor variant & intron variant
AFP10			EQ963478	2252148	G	A	stop gained
**Indels**							
**Isolate**	**Annotation**	**Gene_id**	**CHR**	**SITE**	**REF**	**ALT**	**Type**
AFP10	aflY/hypA/hypP	AFLA_139150	EQ963478	2,187,887	CTGG	TTGA	stop gained
AFP2	aflI/avfA/cytochrome P450 monooxygenase	AFLA_139230	EQ963478	2,204,748	CAGC	TAGG	stop gained
AFP1			EQ963478	2,204,748	CAGC	TAGG	stop gained
AFP10			EQ963478	2,204,748	CAGC	TAGG	stop gained
AFP4			EQ963478	2,204,748	CAGC	TAGG	stop gained
AFP8			EQ963478	2,204,748	CAGC	TAGG	stop gained
AFP11			EQ963478	2,204,748	CAGC	TAGG	stop gained
AFP2	aflL/verB/desaturase/P450 monooxygenase	AFLA_139250	EQ963478	2,205,720	CACTGAGCTGGCCCC	GATTGACCTGGGCGCA	frameshift variant & missense variant
AFP1			EQ963478	2,205,720	CACTGAGCTGGCCCC	GATTGACCTGGGCGCA	frameshift variant & missense variant
AFP4			EQ963478	2,205,720	CACTGAGCTGGCCCC	GATTGACCTGGGCGCA	frameshift variant & missense variant
AFP8			EQ963478	2,205,720	CACTGAGCTGGCCCC	GATTGACCTGGGCGCA	frameshift variant & missense variant
AFP11			EQ963478	2,205,720	CACTGAGCTGGCCCC	GATTGACCTGGGCGCA	frameshift variant & missense variant
AFP10	aflN/verA/monooxygenase	AFLA_139280	EQ963478	2,211,911	ATACA	GTACC	splice donor variant & splice region variant & synonymous variant & intron variant
AFP10	aflT/aflT/transmembrane protein	AFLA_139420	EQ963478	2,250,339	CTTGATA	TTTAATG	stop gained
				2,250,372	AAGAGAGAGAGAGAGAGAGAAAGAAAGAAGAAT	AAGAGAGAGAGAGAGAGAGAGAAAGAAAGAAGAAG	frameshift variant & missense variant
AFP10	aflU/cypA/P450 monooxygenase	AFLA_139430	EQ963478	2,251,958	TGA	TAGA	frameshift variant
				2,251,976	GAATTTCA	GATGGTTCA	frameshift variant & stop gained

Isolate = *Aspergillus flavus* isolate from Pakistan, annotation = alternate gene name, gene ID = gene identifier relative to the *A. flavus* NRRL 3357 reference genome, Chr = *A. flavus* NRRL 3357 scaffold identifier, Site = position in the *A. flavus* NRRL 3357, Ref = reference allele, Alt = Alternate allele in isolate, type = SnpEff predicted type of variant.

## Data Availability

Not applicable.

## Supplementary Materials

The following are available online at www.mdpi.com/article/10.3390/toxins14020117/s1, Figure S1: High-performance liquid chromatography (HPLC) chromatograms of Aflatoxin B1 (AFB1). (A) Combined HPLC chromatograms for high producers AFP1 ~ AFP6. (B) HPLC chromatograms for high producers AFP1 ~ AFP6. (C) HPLC chromatograms for select medium and low producers, Table S1: Read mapping statistics for 12 AFP genomes with the *A. flavus* NRRL 3357 reference genome.
