# Supplementary material for: Characterization of 260 Isolates of Aspergillus Section Flavi Obtained from Sesame Seeds in Punjab, Pakistan
Source: Toxins (Basel). 2022 Feb 4;14(2):117. doi: 10.3390/toxins14020117 (PMC8876583; doi:10.3390/toxins14020117)
Supplement: Supplementary file 1 [file toxins-14-00117-s001.zip › toxins-1575564-supplementary.pdf]

Supplementary Materials: Characterization of 260 isolates *Aspergillus* section Flavi obtained from Sesame Seeds in Punjab, Pakistan

Maryam Ajmal, Ahmad Alshannaq, Heungyun Moon, Dasol Choi, Abida Akram, Brian Gagosh Nayyar, John G. Gibbons and Jae-Hyuk Yu

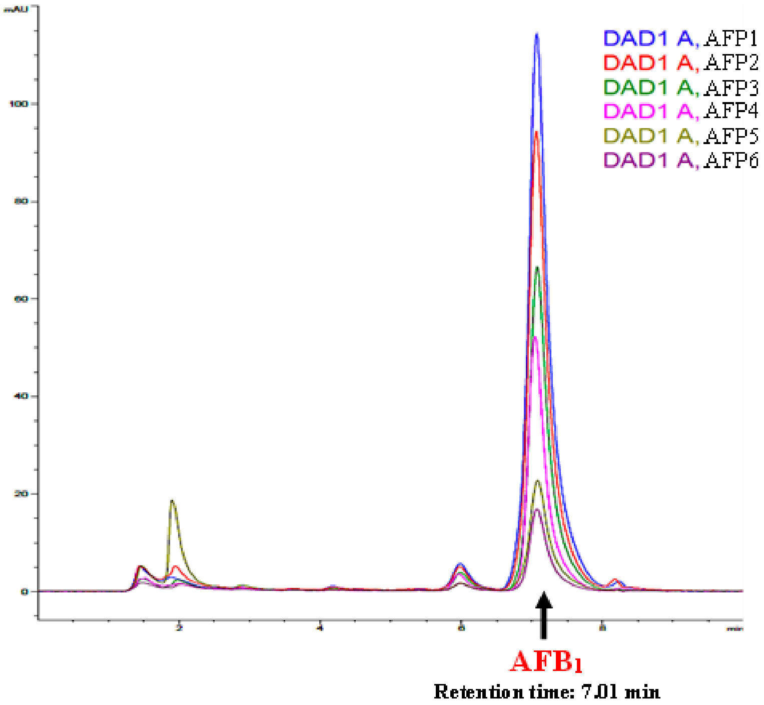

(A)

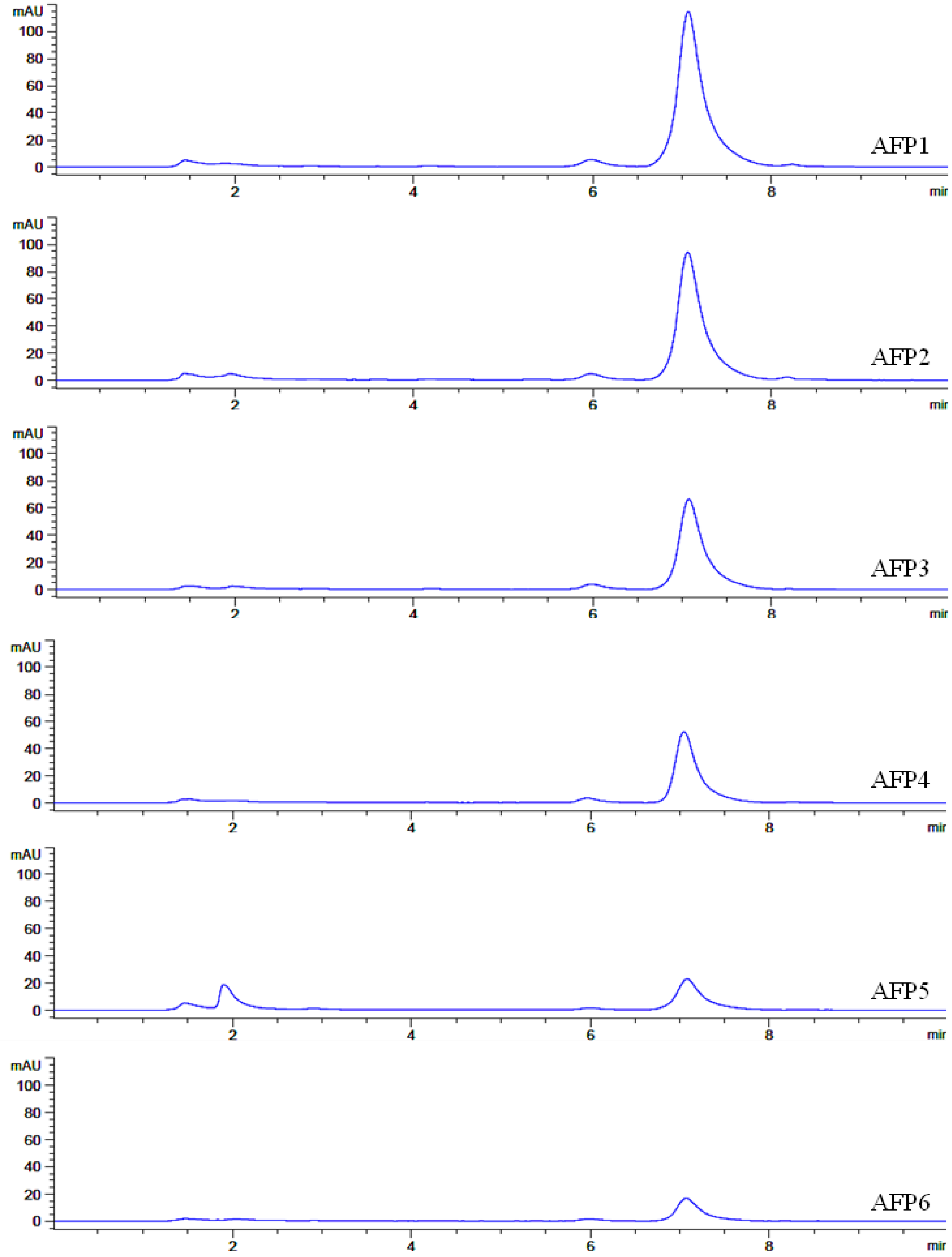

(**B**)

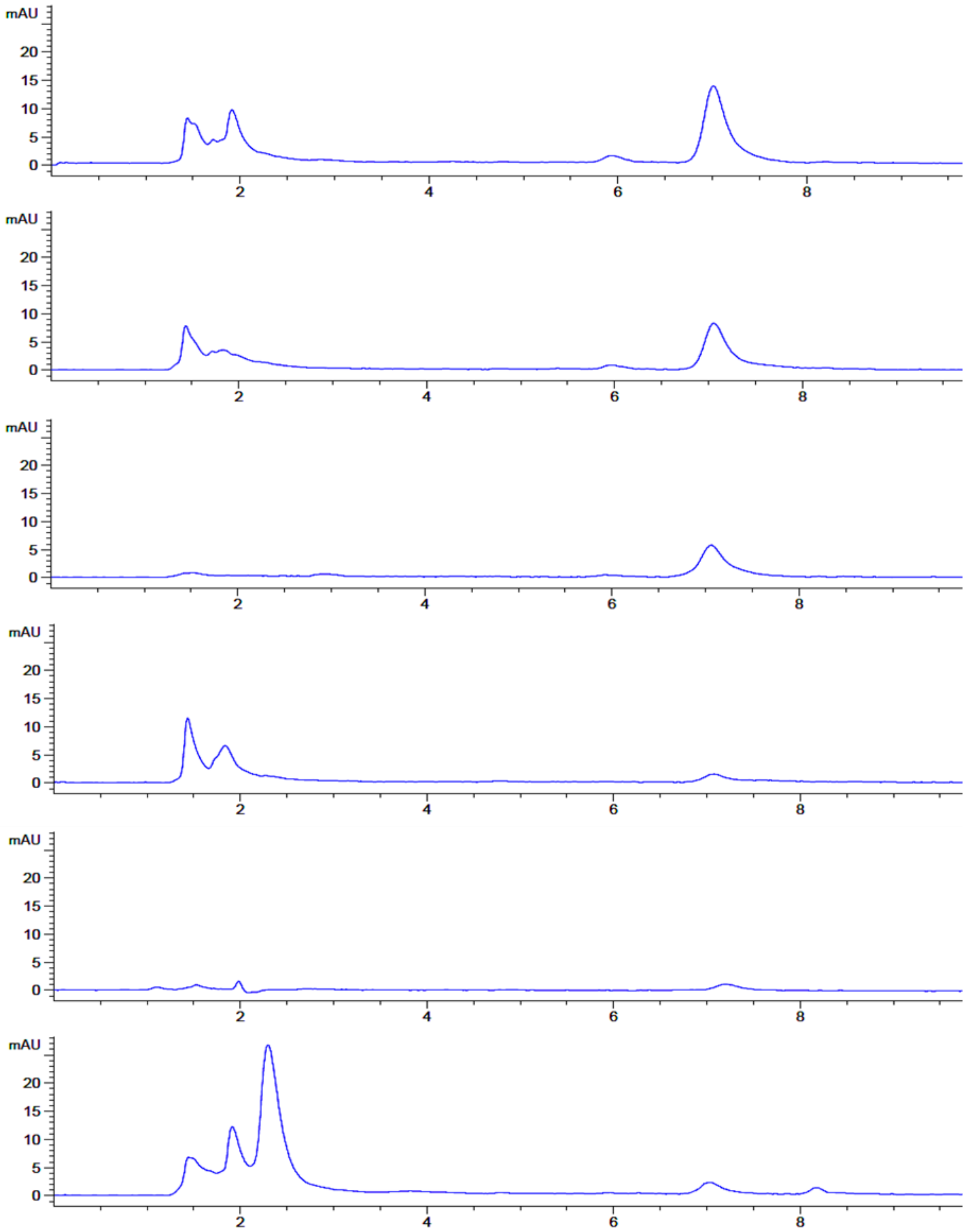

(C)

Figure S1. High-performance liquid chromatography (HPLC) chromatograms of Aflatoxin B1 (AFB1). (**A**) Combined HPLC chromatograms for high producers AFP1 ~ AFP6. (**B**) HPLC chromatograms for high producers AFP1 ~ AFP6. (**C**) HPLC chromatograms for select medium and low producers.

Table S1. Read mapping statistics for 12 AFP genomes with the *A. flavus* NRRL 3357 reference genome.

Sample	Filtered Read Pairs	% Mapped
AFP1	3,895,395	94.05
AFP2	3,341,222	96.51
AFP3	3,443,544	97.04
AFP4	3,906,577	96.2
AFP5	3,837,430	97.2
AFP6	3,424,221	96.83
AFP7	4,273,511	96.88
AFP8	4,828,181	96.49
AFP9	3,628,050	96.7
AFP10	3,461,426	95.8
AFP11	3,332,781	96.55
AFP12	3,282,842	96.07
